# Role of peptide–cell surface interactions in cosmetic peptide application

**DOI:** 10.3389/fphar.2023.1267765

**Published:** 2023-11-13

**Authors:** Bingwei He, Feifei Wang, Liping Qu

**Affiliations:** ^1^ Yunnan Botanee Bio-Technology Group Co, Ltd, Kunming, Yunnan, China; ^2^ Yunnan Yunke Characteristic Plant Extraction Laboratory Co, Ltd, Kunming, Yunnan, China; ^3^ Shanghai Jiyan Biomedical Development Co, Ltd, Shanghai, China

**Keywords:** cosmetic peptides, targeting peptides, anti-oxidative, anti-aging, chondroitin sulfate

## Abstract

Cosmetic peptides have gained popularity in a wide range of skincare products due to their good biocompatibility, effective anti-oxidative properties, and anti-aging effects. However, low binding between peptides and the cell surface limits the efficacy of functional peptides. In this study, we designed two novel targeting peptide motifs to enhance the interaction between cosmetic peptides and the cell surface, thereby improving their performance for skin health. To achieve this, we optimized the well-known peptide tripeptide-1 (GHK) by separately grafting the integrin αvβ3-binding motif RGD and the chondroitin sulfate (CS)-binding motif sOtx2 onto it, forming two chimeric targeting peptides, RGD-GHK and sOtx2-GHK. Comparative analysis showed that both RGD-GHK and sOtx2-GHK exhibited superior anti-oxidative and anti-apoptotic effects compared to the non-targeting peptide, GHK. Furthermore, RGD-GHK demonstrated exceptional anti-aging activity, and its potential for promoting wound healing and repairing the skin barrier was evaluated *in vitro* using cells and skin models. *In vitro* permeation and *in vivo* adsorption testing confirmed that RGD-GHK achieved a high local concentration in the skin layer, initiating peptide effects and facilitating *in vivo* wound healing, while maintaining excellent biocompatibility. The enhancement of signaling cosmetic peptides can be attributed to the specific interaction between the binding motif and cell surface components. Consequently, this targeting peptide holds promising potential as a novel functional peptide for application in cosmetics.

## Graphical Abstract

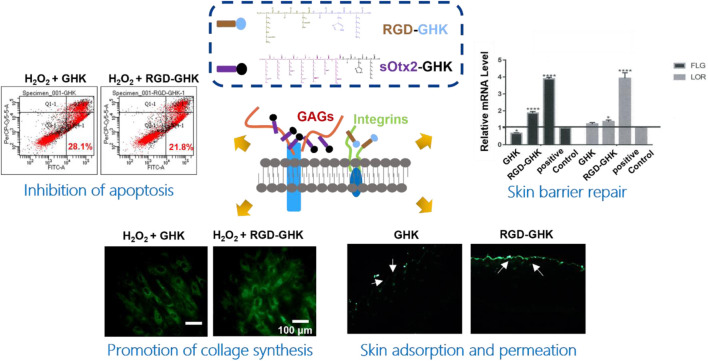

## Introduction

Aging is a natural process in human life, indicating the gradual decay of organisms. Individual factors like genes, race, and diet affect the time and degree of aging (Noren Hooten et al., 2022). However, as individuals age and are exposed to environmental stressors such as air pollutants, ultraviolet (UV) radiation, smoking, and dietary factors, the levels of reactive oxygen species (ROS) increase, leading to inflammation, subsequent protein and lipid peroxidation, the collapse of the defense system, and gene mutations, ultimately accelerating skin cellular pigmentation, senescence, apoptosis, and potentially leading to the development of skin diseases (Martic et al., 2022). Moreover, a substantial reduction in the amount and quality of collagen results in age-related deterioration of skin elasticity and firmness (Bonté et al., 2019). Therefore, attenuation of excessive ROS-induced cellular senescence, apoptosis, and extracellular matrix (ECM) synthesis, especially involving collagen, elastin, and glycosaminoglycans (GAGs), proves efficacious in the advancement of anti-aging therapeutics (Kammeyer and Luiten, 2015).

Bioactive peptides, typically consisting of 3–30 amino acids (AAs), are derived from natural proteins. These peptides serve as signal molecules to trigger cascade reactions or as substrates involved in biological activities (Zhang and Falla, 2009; Aguilar-Toalá et al., 2019). In the last 50 decades, the application of bioactive peptides has been continuously extended in the field of cosmetics. Cosmetic peptides typically contribute to the augmentation of skin cell viability and proliferation, the reduction of skin pigmentation, the mitigation of tissue inflammation, the enhancement of skin barrier functionality, and the provision of support to the skin (Husein el Hadmed and Castillo, 2016). In general, cosmetic peptides are commonly classified according to their mechanisms of action, encompassing signal peptides, carrier peptides, neurotransmitter inhibitor peptides, and enzyme inhibitor peptides. Signal peptides, exemplified by tripeptide-1 and palmitoyl pentapeptide-4, trigger a downstream signaling cascade (Pickart et al., 2015a). Carrier peptides are responsible for transporting microelements, such as Cu and Mg, into cells to maintain essential cellular activities, for example, copper-binding tripeptide (GHK-Cu) (Pickart et al., 2015b). Neurotransmitter inhibitor peptides inhibit acetylcholine (Ach) release at the neuromuscular junction and have a curare-like effect, such as acetyl hexapeptide-8. Enzyme inhibitor peptides directly or indirectly impede the activity of enzymes, while soybean peptide inhibits the formation of proteinase. Cosmetic peptides possess favorable biocompatibility and bioactivities, exhibit low immunogenicity, and operate through well-defined mechanisms. However, low binding affinity between peptides and the cell surface results in insufficient concentrations of functional peptides, thereby constraining the effectiveness of cosmetic peptides in various skincare products (Gorouhi and Maibach, 2009; Pai et al., 2017). Several methods for peptide modification are available, such as introducing hydrophobic groups or conjugating fatty acid chains to functional peptides. These modifications have been shown to enhance the cell membrane adsorption, penetration, and efficacy of peptides; however, this may reduce peptide solubility and increase cytotoxicity. Additionally, various cell surface molecules, including lipids, proteins, and glycosaminoglycans, have been confirmed as targets for peptide–cell membrane interactions. Consequently, a cellular component-dependent targeting peptide modification approach might offer a moderate and reliable method for the improvement of cosmetic peptides.

Integrins, the cell adhesion molecules, are expressed in the cell surface and extracellular matrix (ECM). Integrins comprise a family of over 24 noncovalent, transmembrane heterodimers and have different ligand-binding specificities (Albelda and Buck, 1990; Kadry and Calderwood, 2020). Integrins exert a significant influence on a diverse array of signal transduction cascades that regulate crucial cellular processes, such as cell survival, proliferation, migration, cell–cell and cell–extracellular matrix adhesions, and interactions. Generally, the α-chain interacts with specific signaling molecules, while the β-chain is responsible for intracellular signal transduction. Integrin αvβ3 is made of the αv and β3 chains and is expressed on skin cells, endothelial cells, macrophages, neutrophils, melanoma cells, and most cancer cells (Lygoe et al., 2004; Zoppi et al., 2018). The binding of αvβ3 to extracellular matrix ligands such as FN (fibronectin), VN (vitronectin), and LN maintains the integrity of capillaries (Qin et al., 2013; Fu et al., 2016; Liu et al., 2016). In addition, accumulating evidence suggests that a critical integrin recognition motif, Arg–Gly–Asp (RGD), might potentially bind within a crevice formed by the β-propeller domain of the αv chain and the βA domain of the β3 chain, triggering endocytosis. A number of studies have documented the high expression of αvβ3 in several cancer cells for cell adhesion and migration (Sun et al., 2017). Peptide RGD specifically binds with αvβ3 and results in RGD or RGD-derived peptide-modified vectors delivering anti-cancer drugs, making significant progress in cancer therapy (Nieber et al., 2017). Meanwhile, research results reported that H_2_O_2_ activates p38 MAPK-β3 feedback regulation, which consequently upregulates the expression of the β3 chain, even αvβ3 (Farnoodian et al., 2016). Thus, the upregulation of integrin αvβ3 in skin cells induced by oxidation may serve as a potential therapeutic target for mitigating oxidative damage.

Chondroitin sulfate (CS) is an important polysaccharide belonging to the family of glycosaminoglycans (GAGs) and is ubiquitously expressed on the cell surface. Otx2, a developmental homeoprotein (HP), plays a crucial role in regulating visual cortex plasticity throughout the embryonic, postnatal, and adult stages (Prochiantz and Di Nardo, 2015; Bernard and Prochiantz, 2016). Otx2 is specifically transferred into parvalbumin (PV cells) during the critical period of binocular vision to induce visual cortex onset and closure. The non-autonomous Otx2 activity implies that the specific Otx2-binding sites must be expressed on the surface of PV cells during the plastic period (Sugiyama et al., 2008). It was hypothesized that a 15-amino acid motif located upstream (RKQRRERTTFTRAQL, RK-peptide) of the Otx2 homeodomain is the CS-binding sequence. This assumption has been supported by the fact that injecting synthetic RK-peptide into the visual cortex competitively binds to CS and interferes with the endogenous Otx2 internalization in PV cells. Moreover, the deletion of CS on the cell surface also abolishes the uptake of Otx2 into PV cells (Beurdeley et al., 2012). This study confirmed the role of Otx2 in neurodevelopmental diseases and identified CS as the binding site on the surface of PV cells. The extended studies revealed that RK-peptide is the actual CS-binding sequence of Otx2. In this work, we optimized the RK-peptide by shortening the 15 AAs to seven AAs that maintained specific recognition with CS to obtain a new CS-binding motif, sOtx2.

Here, we have developed two chimeric peptides, namely, RGD-GHK and sOtx2-GHK, composed of an integrin αvβ3-binding or a glycosaminoglycan (GAG)-binding peptide to enhance the specific cell-binding effect, along with GHK for skincare. The incorporation of binding motifs facilitates the localization of GHK near the cell surface and enhances the concentrations of GHK around the membrane receptors to exert cosmetic effects. Compared with GHK alone, both chimeric peptides increased anti-oxidative and anti-apoptotic activities. RGD-GHK also exhibited strong anti-senescent properties in human and mouse embryonic fibroblasts, as well as *in vitro* wound-healing properties and the ability to repair the skin barrier. Notably, RGD-GHK remained present in the epidermis and dermis layers of the skin after washing the skin surface and assisted wound closure *in vivo*, as expected. RGD-GHK presents a novel cell surface-targeting peptide, with promising anti-aging potential for cosmetic applications.

## Materials and methods

### Chemicals

N-Methoxysuccinyl-ala-ala-pro-val-p-nitroanilide (CAS: 70967-90-7) was bought from Sigma Chemical Co. (St. Louis, United States). Sivelestat sodium salt hydrate (S7198, CAS: 201677-61-4) was purchased from Meilun Co. (Dalian). Human neutrophil elastase (30 U/mg) was obtained from Yuanye Bio Co. (Shanghai). p-Nitroanilide (pNA, CAS: 100-1-6) was bought from Aladdin Co. (Shanghai). All other chemicals were of analytical grade and purchased from Sigma.

### Assay of elastase activity

#### Standard curve

The stock solution (0.1 mg/mL pNA) was prepared in 10% DMSO. The standard curve of pNA was made using the different volumes of stock (200, 150, 100, 50, 30, 20, 10, 5, and 1 μL) mixed with 10% DMSO to reach a final volume of 200 μL. The optical density (OD) of each sample was measured at a wavelength of 405 nm.

#### Optimized enzyme concentration

Substrates were dissolved in 10% DMSO to form a final concentration of 100 μM. A measure of 100 μL of different concentrations (10, 5, 2.5, 1.25, 0.625, and 0.3125 U/mL) of elastase solution in 10% DMSO was preheated at 37°C for 5 min, and 100 μL of substrate solution was added to start the reaction at 37°C. The absorbance at 405 nm was monitored at 5-min intervals up to a recording time of 2 h at 37°C. A measure of 200 μL of 10% DMSO was taken as a blank control.

#### Sample test

A measure of 90 μL of 2.5 U/mL elastase solution together with 10 μL of the sample at a certain concentration was preheated at 37°C for 5 min, and then 100 μL of the substrate was added to initiate a 120 min-reaction at 37°C. The OD was read at 405 nm. A measure of 200 μL of 10% DMSO was used as a blank control; 10 μL of Sivelestat solution at 10 mM was prepared as a positive control; and 10 μL of PBS was added as a negative control that represented the maximum enzyme activity. The inhibition rate of elastase was calculated according to the following formula: inhibition (%) = [1 − (OD_sample_ − OD_blank_)/(OD_neg_ − OD_blank_)] × 100%.

### Cell adhesion

HaCaT cells (3.0 × 10^5^) were seeded in sterile cell slides and treated with 200 µM FITC-GHK, FITC-RGD-GHK, and FITC-sOtx2-GHK, respectively, prepared in 500 μL of serum-free DMEM for 4 h at 37°C and 5% CO_2_. After rinsing with PBS, cells were fixed with 4% paraformaldehyde (PFA) for 15 min and stained with 10 μg/mL of Hoechst 33342 and 5 μM of Dil stain solutions (Beyotime) for 10 min for confocal laser scanning microscopy (CLSM) observation (SP8, Leica, Germany). Cells (1 × 10^5^ cells per well) were seeded in a 24-well plate for 24 h and pretreated with FITC-labeled peptide solutions for 4 h at 37°C. Then, cells were washed with PBS and digested using trypsin. The fluorescence intensity of cells was measured via flow cytometry (BD FACSCalibur, USA) to quantitatively analyze the cell-associated peptides.

### Cell viability

Cells (1 × 10^4^ cells per well) were seeded in a 96-well plate to reach approximately 80% confluence. Wells filled with DMEM alone served as positive controls (PCs). The culture medium of sample groups was replaced either by 100 μL of H_2_O_2_ or naked/FITC-labeled peptide solutions at different concentrations for a certain time of incubation, and non-treatment wells acted as negative controls (NCs). Then, the plate solutions were substituted with 100 μL of 10% CCK-8 solution per well and incubated for another 2 h, followed by an absorbance reading taken at 450 nm. The cell survival was determined according to the following formula: cell viability (%) = (OD_sample_ − OD_pc_)/(OD_nc_ − OD_pc_) × 100%.

### Release of lactate dehydrogenase

Lactate dehydrogenase (LDH) activity of the cellular culture was evaluated using an LDH release assay kit (Beyotime). Cells (1 × 10^5^ cells per well) were cultured in a 24-well plate for 24 h to reach approximately 80% confluence and then treated either with peptide solution or peptide-free DMEM (positive control, PC). After 4 h incubation, both the peptide solution and peptide-free culture medium were replaced with 250 μL of 800 µM H_2_O_2_ and incubated for another 2 h. Then, the cells were incubated with 500 µL of the fresh medium instead of H_2_O_2_ for 18 h. Then, 200 µL of the supernatant alone was harvested and mixed with 20 µL of the releasing agent, while the PC medium together with cells was incubated with the equivalent releasing agent. A measure of 200 μL of DMEM alone mixed with 20 µL of the releasing agent was used as a negative control (NC). Then, the solution was incubated at 37°C and 5% CO_2_ for 1 h and centrifuged at 1,000 rpm for 4 min. A measure of 40 μL suspension per well was pipetted into a 96-well plate and mixed with 20 µL of fresh LDH working solution that was prepared according to the manufacturer’s protocol. The plate was briefly vortexed in the dark for 30 min, and the OD values were measured at 490 nm wavelength. LDH release (%) was expressed via the following equation: LDH (%) = (OD_sample_ − OD_nc_)/(OD_pc_ − OD_nc_) × 100%.

### Cell apoptosis assay

The percentage of early-stage apoptosis and late-stage apoptosis and necrosis of cells induced by H_2_O_2_, with or without peptide addition, were determined using an Annexin V-FITC/PI double-staining assay kit (Beyotime). Cells (1×10^5^ cells per well) were seeded in a 24-well plate, pretreated with or without peptide solutions, and incubated for 4 h. The peptide solution or culture medium was replaced with 800 μM of H_2_O_2_ solution and was incubated for 2 h at 37°C. Then, the cells were incubated only with the complete medium for another 18 h. Non-treated groups were taken as controls. Suspended medium (200 µL) and trypsin-disassociated cells were collected and centrifuged at 1,000 rpm for 4 min. The precipitate was resuspended in binding buffer (195 µL) plus Annexin V-FITC (5 µL) and incubated for 5 min, followed by 10-min staining of 10 µL propidium iodide (PI). The non-staining sample was used as a reference. After a gentle mix at room temperature and a dark environment, the ratio of cell death was detected via flow cytometry (FCM). The cell plot was distributed into four quadrants based on Annexin V-FITC and PI axes, representing living cells in quadrant 3 (FITC-/PI-), early-stage apoptotic cells in quadrant 4 (FITC+/PI-), and total late-stage apoptotic and necrotic cells in quadrant 1 (FITC+/PI+). Cysteine–aspartic acid-specific protease-3 (caspase-3) plays a major role in triggering the apoptotic process, which is commonly cleaved by poly (ADP-ribose) polymerases (PARP) involved in DNA repair and transcriptional regulation during apoptosis. Thus, caspase-3 activity has been suggested to be an index of apoptosis. We used a highly cell-permeable and non-fluorescent caspase-3 substrate, DEVD dye consisting of peptide DEVD conjugated to a fluorogenic DNA-binding dye. The free dye was inclined to bind to DNA in the nucleus and emitted strong green fluorescence when DEVD dye was broken down by caspase-3. The fluorescence intensity was measured by FCM, which represented the activity of caspase-3.

### Reactive oxygen species assay

HaCaT cells (10^5^ cells per well) were seeded in a 24-well plate for 24 h and pretreated with peptide solutions for another 4 h. The cells were treated with 800 μM H_2_O_2_ for 2 h at 37°C and 5% CO_2_. The level of reactive oxygen species was quantified immediately using a ROS detection kit (Beyotime) and FCM. Briefly, cells were washed twice with serum-free DMEM and incubated with 1,000 × diluted DCFH-DA, which was hydrolyzed to non-fluorescent DCFH inside the cell. DCFH is oxidized to fluorescent DCF (dichlorofluorescein) by reaction with ROS. Thus, the relative cellular ROS level could be represented by the relative DCF fluorescence intensity analyzed by FCM.

### Quantification of hydroxyproline

Hydroxyproline (Hyp) is a non-essential amino acid found in collagen (accounting for approximately one-third of body proteins in humans and other animals) and plays a crucial role in collagen synthesis and thermodynamic stability of the triple-helical conformation of collagen and associated tissues (Srivastava et al., 2016). The change in Hyp could be a biochemical marker to understand the pathogenesis of several diseases. Poor wound healing leads to a decreased level of Hyp. Quantification of Hyp, to some extent, reflected the content of collagen. Briefly, 1 × 10^5^ HFF-1 cells per well were seeded in a 12-well plate for 24 h. Then, the cells were treated with peptide solution for 4 h, followed by a replacement of 400 µM H_2_O_2_ for 2 h. The concentration of Hyp, which represented the collagen content of HFF-1, was examined using a Hyp assay kit (Nanjing Jiancheng Bioengineering Research Institute, Nanjing, China) according to the manufacturer’s instructions. The absorbance of samples was read at 550 nm wavelength.

### Enzyme-linked immunosorbent assay

HFF-1 cells (1 × 10^5^ cells per well) were seeded in a 24-well plate for 24 h. The cells were pretreated with 500 µL of peptide solution at 200 μg/mL for 4 h, followed by the replacement of 400 µM of H_2_O_2_ for 2 h. Then, the cells were incubated with fresh complete medium for another 18 h. Matrix metalloproteinase-1 (MMP-1) in the supernatant was detected according to the procedure of the ELISA kit (Xinbosheng, China).

### Quantitative real-time polymerase chain reaction

HFF-1 cells (1 × 10^5^ cells per well) were seeded in a 12-well plate for 24 h. The following treatments of peptide and H_2_O_2_ are the same as those described in the *Enzyme-linked immunosorbent assay* (ELISA) section. The supernatant was collected for ELISA, while the mRNA of the cell precipitate was extracted using a GeneJET RNA purification kit (Thermo Fisher Scientific). The total mRNA was quantified using NanoDrop one (Thermo Fisher Scientific) and reverse-transcribed using PrimeScript RT Master Mix (Takara, Shiga, Japan) according to the manufacturer’s protocol (500 µg RNA was reverse-transcribed to cDNA in a final volume of 10 µL). Quantitative real-time polymerase chain reaction (qRT-PCR) was performed in a total volume of 20 µL, including 10 µL of SYBR Green PCR Master Mix (Roche), 2 µL of cDNA template, and 0.4 µL of each primer (10 µM) using a LightCycler 96 instrument (Roche). The standard cycling conditions were 95°C for 10 min, followed by 40 cycles of 95°C for 30 s and 60°C for 30 s. The ratios of the number of candidate genes (MMP-1 and COL-1) to the housekeeping gene were analyzed using LightCycle 96 software to evaluate the relative mRNA level of each candidate. The sequences of primers are listed in Supplementary Table S1.

### Scratch wound assay

Special Ibidi culture inserts (8.4 mm*8.4 mm*5 mm) consist of two reservoirs separated by a 500 µm wall. An insert was placed in one well of a 12-well plate and lightly pressed down to ensure tight adhesion. A measure of 70 μL of cell suspension (2 × 10^5^ HaCaT cells/mL) was added into both chambers, while another 200 µL of cell suspension was filled in the surround of the insert for adhesion overnight. The insert was gently removed creating a gap of approximately 500 µm and then re-incubated in the presence or absence of peptides (200 μg/mL of GHK and RGD-GHK) for 24 h. Treatment with 10,000 IU/mL EGF served as a positive control (PC). Cells were observed under a bright microscope at 0 h and 24 h. Rates of wound closure were photographed and assessed using ImageJ software. The healing rate was calculated according to the following formula: healing rate (%) = {scratched area (0 h) − scratched area (24 h)}/{scratched area (0 h)}.

### Skin barrier repair

Commercial epidermal tissues EpiSkin (L'Oréal Research and Innovation Center, Shanghai, China) is an *in vitro* reconstructed human epidermis (RHE) from normal human keratinocytes and is similar to the *in vivo* human epidermis. EpiSkin is normally cultured on a collagen matrix at the air–liquid interface and could be removed from the nutrient agar and equilibrated in 12-well plates at 37°C and 5% CO_2_ overnight. EpiSkin was treated with 2 mg/mL peptides for 24 h. The non-treated group was used as a control, and cell-penetrating peptide (CPP)-conjugated GHK was used as a positive control. The amphiphilic cell-penetrating peptide (CPP) exhibits potential advantages in terms of enhanced penetration through the skin’s lipid barrier and efficient transportation of GHK to the cellular surface, thereby demonstrating a remarkable efficacy in skin regeneration. The expression of proteins filaggrin (FLG) and loricrin (LOR) was evaluated by immunofluorescence, while the expression of mRNAs FLG and LOR in the skin was measured via qRT-PCR.

### Immunofluorescence staining

HFF-1 cells (1.5 × 10^5^ cells per well) or mouse embryonic fibroblasts (NIH3T3) were grown on sterile cell slides for 24 h till 80% confluence. A measure of 200 μg/mL of RGD-GHK and GHK solutions was prepared with serum-free DMEM, and the cells were treated with the solutions for 4 h. Then, the solutions were replaced with 400 µM H_2_O_2_ and incubated for another 2 h. Then, the fresh culture medium was added to the cells and incubated for another 20 h. Pretreatment without peptides was used as a negative control, while treatment with an anti-aging peptide, acetyl hexapeptide-8 (Wrinklend), was taken as a positive control. The slides were washed three times with PBS, followed by fixation in 500 µL of 4% paraformaldehyde for 15 min at room temperature. After washing with PBS another three times, the cells were permeabilized with 0.5% Triton X-100 for 15 min and washed with PBS repeatedly. The cells were blocked using 1% BSA for 1 h to eliminate non-specific binding. Samples were incubated at 4°C overnight with 100 µL of rabbit monoclonal antibody against type I collagen (COL1A1, 1:100 dilution, CST). Samples were then washed with PBS as described previously and incubated with goat anti-rabbit secondary antibody (1:500 dilution, Beyotime) at room temperature for 1 h in darkness. The cell slides were washed and positioned into the glass microscope slide using clean forceps. Then, a fluorescent anti-quenching agent was applied to seal off the slips for 5 min, and the slips were observed using an upright fluorescence microscope.

### Proteolytic stability assay

To evaluate the proteolytic stability of peptides against chymotrypsin, the peptides were dissolved to 1 mg/mL and incubated with chymotrypsin (1 mg/mL) at 37°C for different times (0, 1, 2, 4, 8, and 16 h). Afterward, the remaining peptides in the samples were analyzed by C18 reversed-phase high-performance liquid chromatography (HPLC) and detected at 220 nm. The mobile phase of HPLC was a mixture of acetonitrile and deionized water containing 0.1% trifluoroacetic acid.

### Blood compatibility

The blood compatibility of peptides was assessed using mouse red blood cells. The procedure involved obtaining red blood cells by centrifuging mouse blood at 2,000 rpm for 5 min, followed by washing them with PBS three times. The red blood cells were then diluted to a final concentration of 5% (v/v). Then, 2 mg/mL of peptides were added to 1 mL of the red blood cell suspension, and the mixture was incubated at 37°C for 1 h. After incubation, the suspension was centrifuged at 2000 rpm for 5 min, and the supernatant was measured at 540 nm using a microplate reader. The positive control consisted of 0.1% Triton X-100, while PBS served as a negative control. Each experiment was repeated five times. The hemolysis percentage was calculated using the following formula: hemolysis (%) = [(OD_sample_ −OD_nc_)/(OD_pc_ − OD_nc_)] × 100%.

### *In vivo* skin penetration assay

Female BALB/c nude mice with an average weight of 20 g were purchased from SLAC Laboratory Animal Co., Ltd. (Shanghai, China). For the skin penetration study, the dorsal skin of each mouse was cleaned using an alcohol pad, and 14 mg of cream containing 0.2% FITC-GHK, FITC-RGD-GHK, or FITC-sOtx2-GHK peptides were smeared uniformly on the skin (1 cm^2^). Each group consisted of three mice. After 24 h, the mice were euthanized and evaluated for peptide adsorption and penetration analysis by histological examination. The harvested skin samples were frozen and sectioned into 4-μm slices, mounted onto glass slides, and examined using a fluorescence microscope (DMi8, Leica, Germany).

### *In vivo* wound healing

In this study, a full-thickness skin defect model using female BALB/c mice weighing 20 g and aged 6–8 weeks was employed to assess the wound-healing capability of GHK and RGD-GHK peptides. The mice were initially anesthetized with pentobarbital (50 mg/kg body weight) administered through intraperitoneal injection. The hair on the back of the mice was then shaved to prepare for surgery. To create the full-thickness circular skin wound, a skin punch with an 8 mm diameter was used. All surgical procedures were conducted under sterile conditions to prevent contamination. Following the creation of the wounds, 14 mg of cream containing 0.2% GHK or RGD-GHK peptides was applied to the wounds every 2 days for a total of five applications. In the control group, a cream without peptides was used. To evaluate the wound closure capability of the peptides, photographs of the wounds were taken at 0, 5, 10, and 15 days after the initial application of the cream. The photographs were then analyzed to determine the extent of wound closure facilitated by the peptides.

### Histological and immunohistochemistry examination of skin wounds

To assess the epidermal regeneration and inflammation in the wound area, tissue samples were collected on the 15th day after the initial application of the cream. The samples were fixed in 4% paraformaldehyde for 1 h, followed by embedding in paraffin. The paraffin-embedded samples were then transected into 4-μm-thick sections. Hematoxylin–eosin (H&E) and Masson’s trichrome staining techniques were performed on these sections to visualize the cellular structures. The stained sections were then photographed and analyzed under a microscope (DMi8, Leica, Germany) to evaluate the extent of epidermal regeneration in the wound area. Additionally, on the 15th day, regenerated skin from the wound site was excised for immunofluorescence staining. Fixed and frozen sections were prepared from the excised skin. The fixed sections were stained with anti-IL-6 antibody (Abcam) and anti-CD31 antibody (Abcam). The stained slides were then observed under an inverted fluorescence microscope (DMi8, Leica, Germany).

### Data statistics

Data were represented as the mean ± standard of at least two independent experiments performed in duplicate or triplicate. Differences were considered at N.S. *p* > 0.05, *p < 0.05, **p < 0.01, and ***p < 0.001 using two-tailed Student’s t-test.

## Results and discussion

### Schemes of cell component-targeted peptides, RGD-GHK, and sOtx2-GHK

**FIGURE 1:**
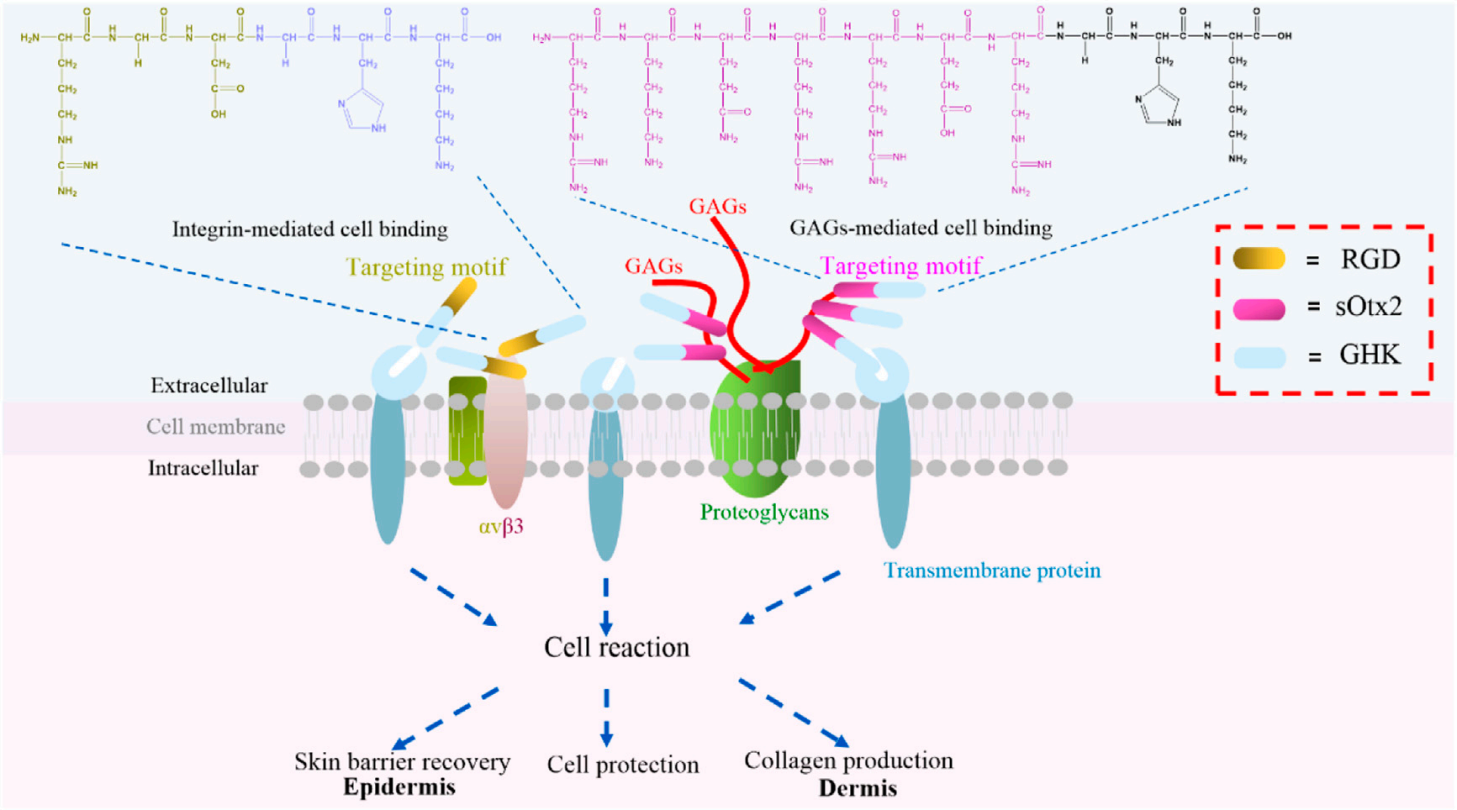
Illustration of two novel targeting peptides, αvβ3-binding and chondroitin sulfate (CS)-binding cosmetic peptides, RGD-GHK (brown and blue bar) and sOtx2-GHK (purple and blue bar).

Tripeptide-1 (GHK), made of three amino acids and derived from collagen Iα2, is a white powder widely used in skincare products. GHK initiates the cellular downstream pathway and exerts bioactivities through binding with cell surface receptors (Pickart et al., 2015a). Based on tripeptide-1, the derived peptide, human copper-binding peptide GHK-Cu, is a tripeptide with high affinity for copper ions, which is also known to have multiple beneficial effects on human health, including skin rejuvenation (Pickart et al., 2015b). Regardless of the mechanism of GHK and GHK-Cu, GHK-Cu also stimulates expression of collagen, elastin, and glycosaminoglycan, which makes it a frequently used cosmeceutical peptide ingredient for supporting the growth and functions of dermal fibroblast (Fu et al., 2015). Furthermore, GHK is usually conjugated with a fatty acid, palmitic acid, to form Pal-GHK to increase stability and penetration into the skin. Thus, a high concentration of Pal-GHK around the cell surface contributes to the delivery of GHK. *In vitro* and *in vivo* studies proved that this combination stimulates the synthesis of collagen and glycosaminoglycans (Wegrowski et al., 1992; Maquart et al., 1993). Although various changes made certainly improve the delivery of GHK (Li et al., 2015), fatty acid modification complicated the process of peptide synthesis and decreased the solubility of the peptide. Plenty of biomolecules, such as glycosaminoglycans (GAGs), integrins, and proteoglycans, are ubiquitously expressed on the cell surface. These molecules are responsible for cell–cell communication, cell–cell interaction, and cell–cell adhesion, which are indispensable during life activities (Crocker and Feizi, 1996). Here, we designed two cell component-targeted cosmetic peptides, and an integrin αvβ3-targeted motif RGD and a GAG (chondroitin sulfate, CS)-targeted motif sOtx2 (sequence: RKQRRER) were synthesized and grafted to the N-terminus of GHK to form two novel cosmetic peptides, RGD-GHK and sOtx2-GHK (Figure 1), respectively. The quality controls of GHK and these two targeting peptides are shown in Supplementary Figure S1. RGD-GHK and sOtx2-GHK were envisaged to increase the concentration of tripeptide-1 at the cell surface and enhance the efficacy of GHK.

### Inhibition of elastase activity

**FIGURE 2:**
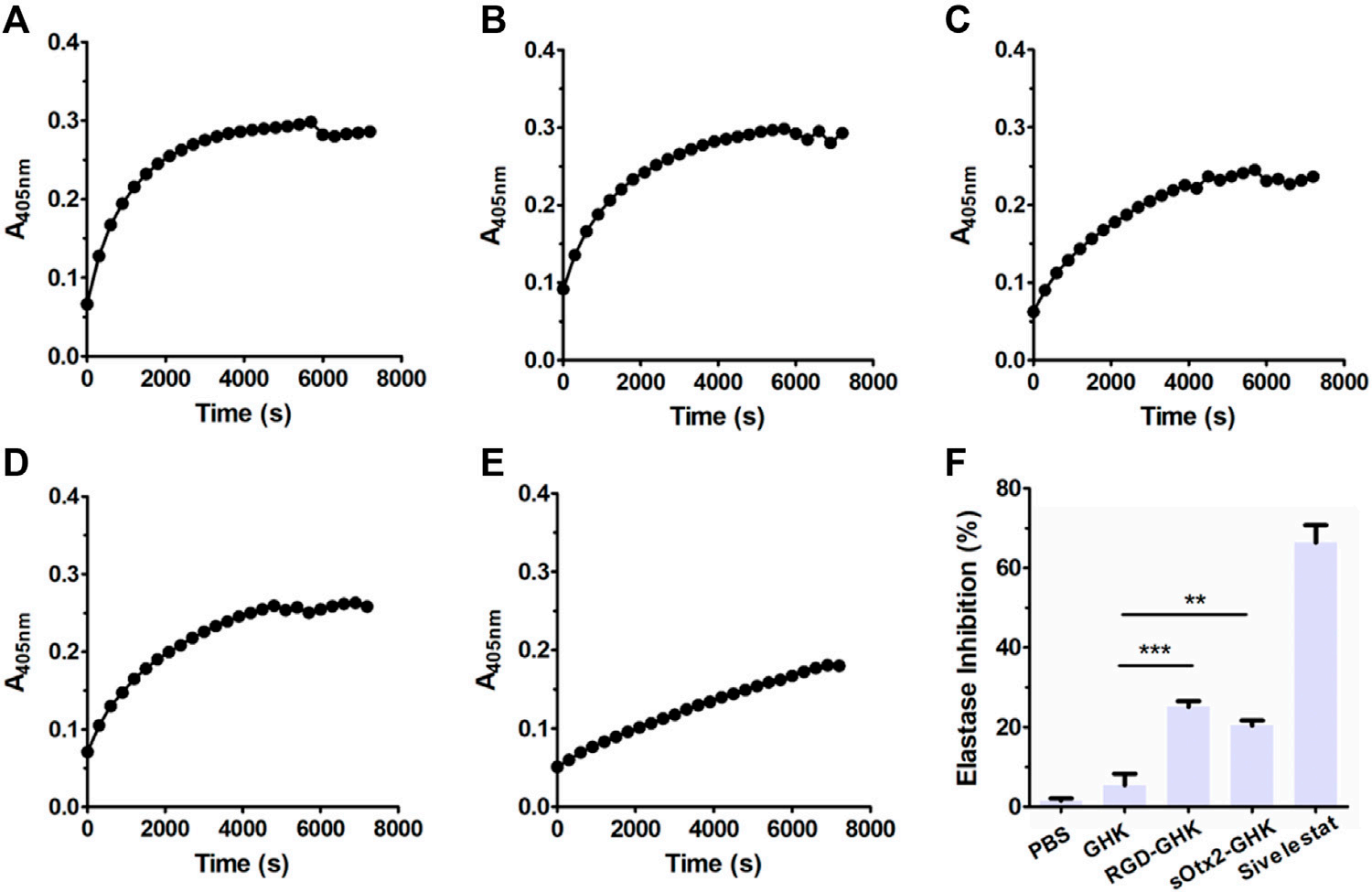
Elastase inhibition curve of PBS **(A)**, GHK **(B)**, RGD-GHK **(C)**, and sOtx2-GHK **(D)** at a concentration of 200 μg/mL and 250 μM of Sivelestat **(E)** recorded for each 5-min interval within 2 h; statistics of complete inhibition rate of inhibitors at 2 h time point **(F)**.

The skin is the largest organ in the human body. The dermis is the major constituent of the skin and is composed primarily of collagen, elastin, and glycosaminoglycans, which provide the skin its elasticity and flexibility (Huang et al., 2022). The main component collagen plays a key role in the structure and integrity of the organism. The expression of enzymes such as collagenase, elastase, and hyaluronidase is stimulated, leading to the deterioration of collagen, elastin, and other components of the dermal extracellular matrix (Freitas et al., 2020). Here, we measured the inhibition rates of GHK and two targeting peptides on elastase. The reaction curves of elastase, either in the presence or absence of peptides, during 2 h are recorded in Figures 2A–E. Figure 2F suggests that the results of the elastase inhibition rate of targeting peptides are very commendable, approximately 25% for RGD-GHK and 20% for sOtx2-GHK produced. Both significantly suppressed the elastase activity compared with the non-targeting peptide, GHK. Sivelestat is used as a commercial elastase inhibitor, which achieved approximately 65% inhibition of elastase.

### Cell adhesion

**FIGURE 3:**
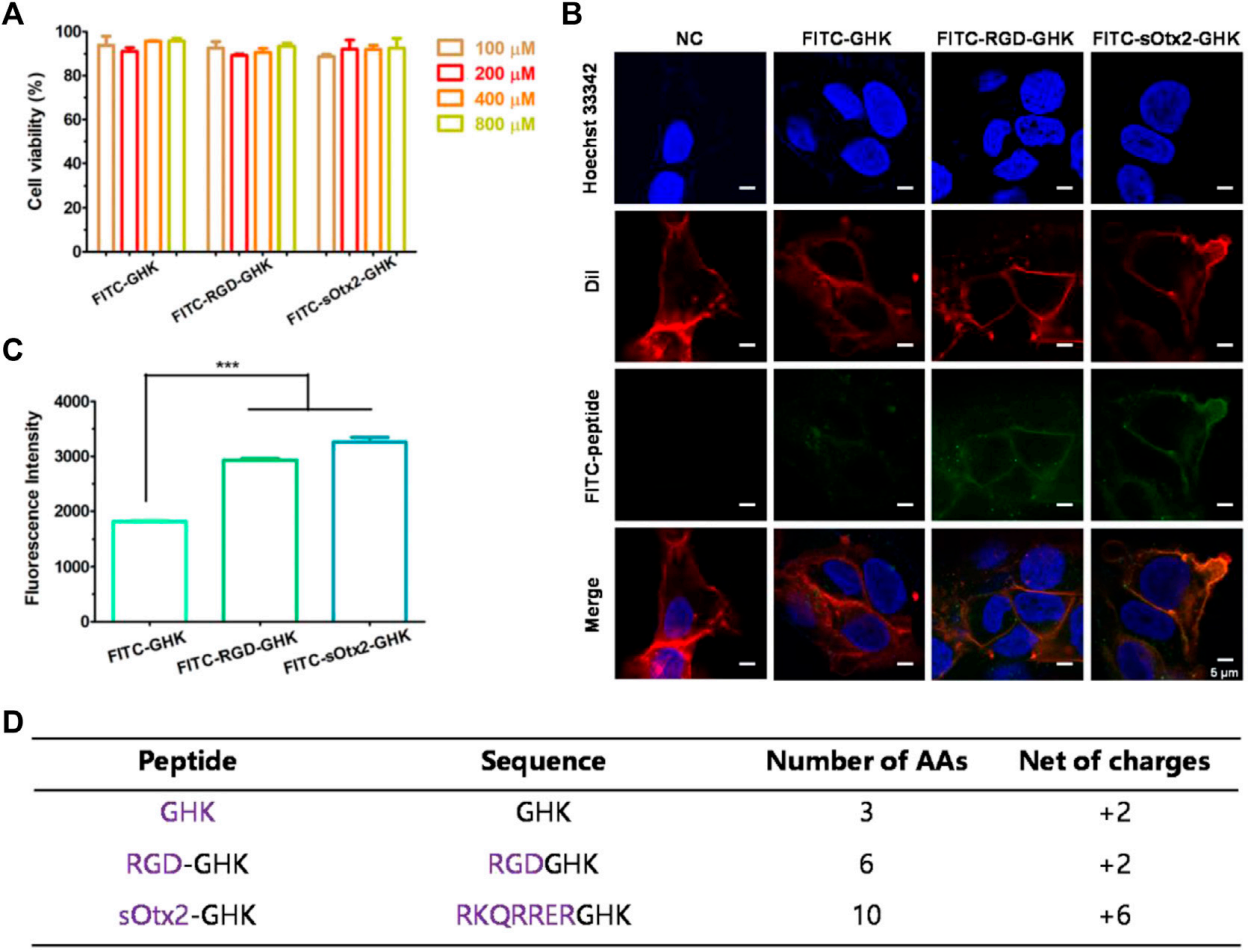
Deposition of FITC-labeled peptides on HaCaT cells. Cell viability of FITC-labeled peptides at different concentrations on HaCaT **(A)**. Confocal images of the corresponding peptide and cell membrane colocalization after 4 h of incubation. The green channel represents FITC-peptides. Nuclei and cell membranes were stained with Hoechst (blue) and Dil (red) **(B)**. Results of flow cytometry (FCM) values of cells after incubation with FITC peptides for 4 h **(C)**. Table represents the properties of GHK and two modified GHK variants **(D)**.

The estimable results of elastase inhibition rates produced by chimeric peptides encourage us to evaluate the skin protection and skin anti-aging properties of targeting peptides in skin cells. First, we estimated the cellular localization of peptides. Cells were incubated with the fluorescent probe (FITC)-labeled peptides at different concentrations for 4 h with minimal cytotoxicity (Figure 3A). In Figure 3B, the high fluorescence intensity of FITC-sOtx2-GHK and FITC-RGD-GHK were observed by CLSM, while FITC-GHK exhibited only modest fluorescence, and peptides colocalized with the cell membrane. FACS confirmed that the results are consistent with the observation (Figure 3C). It is reasonable to believe that sOtx2-GHK with six net positive charges and RGD-GHK with recognition contribute to the overall cell surface adsorption of GHK peptide. However, GHK with two net positive charges leads to lower cell membrane adsorption than the two chimeric peptides (Figure 3D).

### Targeting peptides protected keratinocytes against H_2_O_2_ damage

**FIGURE 4:**
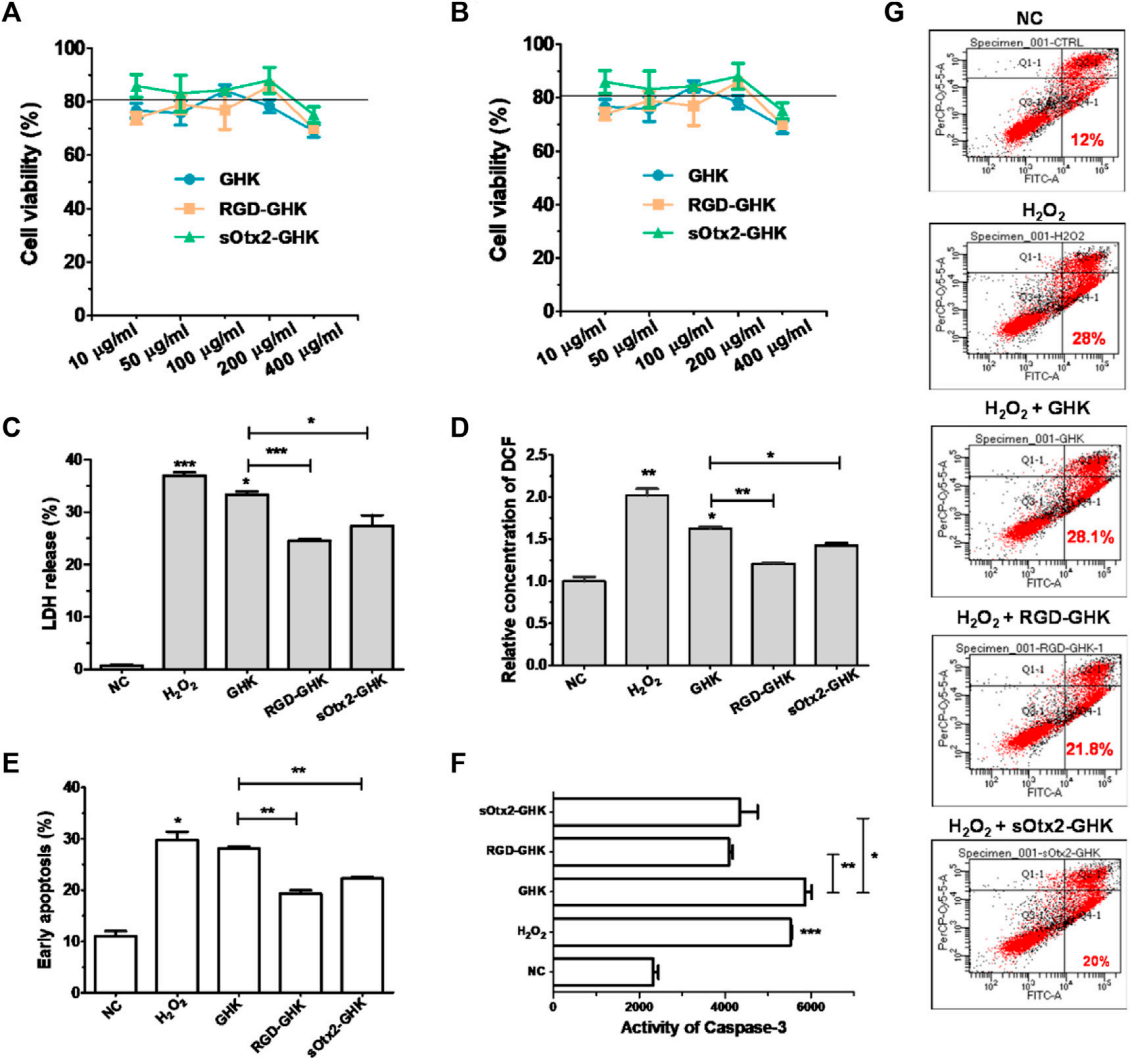
Protection effect of peptides against oxidative damage on HaCaT. Cytotoxicity of GHK, RGD-GHK, and sOtx2-GHK in HaCaT **(A)** and HFF-1 **(B)**. Cells were incubated with various concentrations of the peptides for 24 h, and the cell viability was determined via the CCK-8 assay. Cells were pretreated with peptides (200 μg/mL) for 4 h and exposed to 800 μM H_2_O_2_ for another 2 h; then, the H_2_O_2_ solution was replaced by the fresh medium, and the cells were incubated overnight **(C, D)**. Subsequently, the percentages of LDH leakage in culture supernatants were measured **(C)**. The level of reactive oxygen species (ROS) was quantified immediately after H_2_O_2_ treatment using a membrane-permeable probe DCFH-DA and FCM **(D)**. The cells were digested and analyzed for apoptosis by FCM **(E–G)**. Distributions of cell populations stained with FITC-Annexin V and/or PI **(G)**, the percentage of early apoptosis **(E)**, and the activities of caspase-3 **(F)** induced by H_2_O_2_ and peptides.

We tested the cytotoxicity of peptides at different concentrations on two human skin cell lines that were mainly distributed in the epidermis and dermis layers. In Figures 4A, B, GHK and the two targeting peptides exhibited negligible cytotoxicity in human keratinocyte (HaCaT) and human fibroblast (HFF-1) at concentrations up to 200 μg/mL that was used for the following experiments.

Tripeptide-1 protects skin cells against oxidative stress and enhances collagen and glycosaminoglycan (GAG) production. Thus, we used an H_2_O_2_-damaged skin cell model treated with different concentrations of H_2_O_2_ at various times. Supplementary Figure S2 shows that HaCaT cells had an apparent cytotoxic effect with the treatment of 1,600 μM of H_2_O_2_, while HFF-1 showed obvious cytotoxicity at 800 μM of H_2_O_2_ after 2 h incubation. In this case, HaCaT cells exposed to 800 μM of H_2_O_2_ and HFF-1 cells exposed to 400 μM of H_2_O_2_ for 2 h incubation were applied in the oxidative stress models.

The intracellular lactate dehydrogenase (LDH) activity of living cells is relatively constant during the culture, while impaired cells lead to LDH leakage to the culture medium (Cho et al., 2008). Thus, the LDH level of the culture medium can represent a good qualitative and quantitative indicator of cellular death. Spontaneous LDH released from HaCaT cells after incubation with 800 μM of H_2_O_2_, either in the presence or absence of peptides, was determined by using the spectrophotometric method. H_2_O_2_ caused approximately over 37% of LDH leakage, while RGD-GHK and sOtx2-GHK attenuated H_2_O_2_-induced LDH release by 12% and 10%, respectively, as shown in Figure 4C. The results illustrated that the two targeting peptides exerted strong protective effects on HaCaT from oxidative damage. Furthermore, Figure 4D confirmed that H_2_O_2_ increased the content of cellular ROS, while both targeting peptides obviously reduced the fluorescence intensity of the cellular ROS probe compared to that of GHK, especially RGD-GHK inhibited approximately 40% of ROS production. Consequently, the two chimeric peptides achieved more efficacy in protecting the cell against oxidative damage than GHK.

Severe oxidative stress can seriously impact cell viability, causing early apoptotic cells and cell death. Since targeting peptides exhibited the anti-oxidative ability in previous experiments, we further studied their anti-apoptotic effect. In Figure 4G, H_2_O_2_ induced 28% early apoptosis, while RGD-GHK and sOtx2-GHK prominently decreased the ratio of early apoptosis to approximately 21% (Figure 4E). Caspase-3 (cysteinyl aspartate-specific proteinase-3) is an important proteolytic enzyme responsible for the execution of apoptosis. Once activated, caspase-3 initiates the cleavage of a downstream substrate, such as nuclear poly (ADP-ribose) polymerase (PARP), which facilitates cellular disassembly and serves as a biomarker of cell apoptosis. Figure 4F illustrates that both targeting peptides significantly inhibited caspase-3 activity since decreasing fluorescence intensities of caspase-3 substrates were observed. Therefore, targeting peptides took part in cell anti-apoptosis.

### Targeting peptides promoted collagen synthesis

**FIGURE 5:**
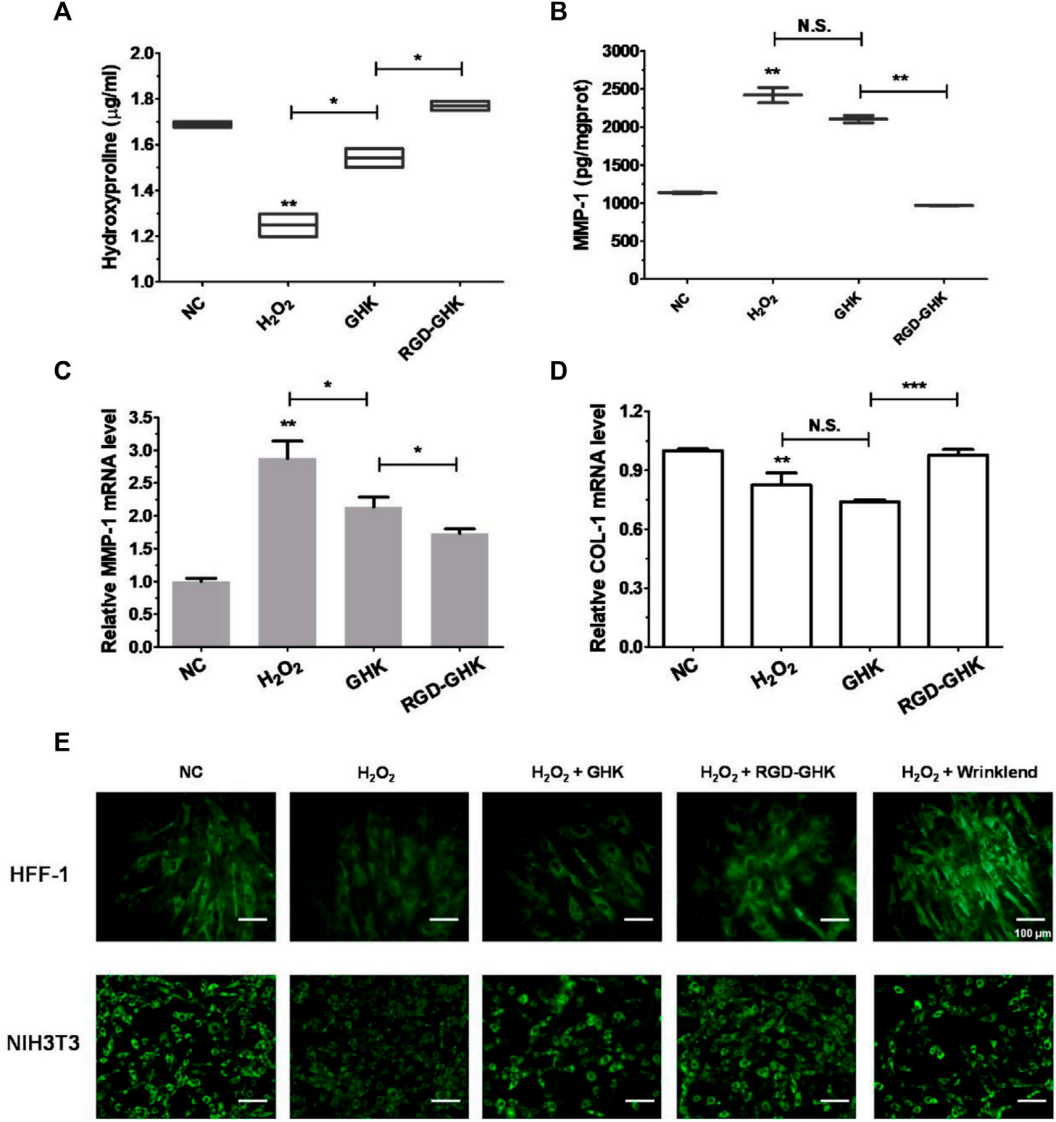
Anti-senescent effect of peptides on H_2_O_2_-induced HFF-1 and NIH3T3. Quantification of hydroxyproline by colorimetric assay on HFF-1 **(A)**. Cells were treated with peptides at concentrations of 200 μg/mL 4 h prior to incubation with H_2_O_2_ (400 μM) for 2 h, then H_2_O_2_ was replaced with the fresh medium, and cells were continuously incubated for 18 h on HFF-1 **(B–D)**. MMP-1 expression in the supernatant was detected by enzyme-linked immunosorbent assay (ELISA) **(B)**, while cells were collected and lysed for qRT-PCR analysis (β-actin expression was used as an internal control) **(C, D)**. Immunofluorescence staining of marker COL-1 of HFF-1 and NIH3T3 **(E)**. Cells were grown on sterile cell slides overnight and pretreated with 200 μg/mL of RGD-GHK or GHK for 4 h and then incubated with 400 µM H_2_O_2_ for 2 h. Then, cells were cultured with the fresh culture medium for another 20 h. Wrinklend served as a positive control. The slides were then fixed and stained with 100 µL of type I collagen antibody (COL1A1, 1:100 dilution, CST), followed by incubation with goat anti-rabbit secondary antibody (1:500 dilution, Beyotime). The cell slides were observed using an upright fluorescence microscope.

Both targeting peptides exhibited anti-oxidative and anti-apoptotic properties. We quantified the trade-offs between efficiency and cost, considering RGD-GHK for the following experiments. Since tripeptide-1 has an anti-aging effect, we compared the anti-senescent effect of RGD-GHK and GHK. We first evaluated the collagen content via hydroxyproline determination measured by colorimetric assay. The level of collagen hydrolysis product hydroxyproline suggested that H_2_O_2_ accelerated collagen hydrolysis, while RGD-GHK prevented more collagen from degradation compared with the action of GHK, as shown in Figure 5A. Furthermore, Figures 5B, C suggest that RGD-GHK evidently suppressed the protein and mRNA level of matrix metalloproteinase-1 (MMP-1) *in vitro*, which catalyzes the cleavage of collagen. RGD-GHK also enhanced the COL-1 mRNA level, which encodes collagen type I, as shown in Figure 5D. COL-1 fibrils are the abundant component of dermal extracellular matrix (ECM) which confers strength and resiliency on the skin. During aging, ECM interactions become disrupted due to the fragmentation of collagen fibrils. COL-1 of the human fibroblasts HFF-1 and mouse embryonic fibroblasts NIH3T3 was stained using an anti-COL-1 antibody and observed via fluorescent microscopy. Figure 5E reveals that the fluorescent intensity was reduced after H_2_O_2_ treatment, while incubation with targeting peptide RGD-GHK recovered COL-1 expression. Wrinklend, an acetyl hexapeptide-8, is well-known for its anti-aging abilities. It was used as a positive control that significantly suppressed COL-1 degradation and maintained skin health. The results directly indicated that the targeting motif RGD-binding peptide RGD-GHK almost totally suppressed the collagenase (MMP-1) expression, resisted H_2_O_2_-induced COL-1 degradation, and simultaneously promoted collagen synthesis compared with GHK alone for cell anti-aging performance.

### Skin repair *in vitro*

**FIGURE 6:**
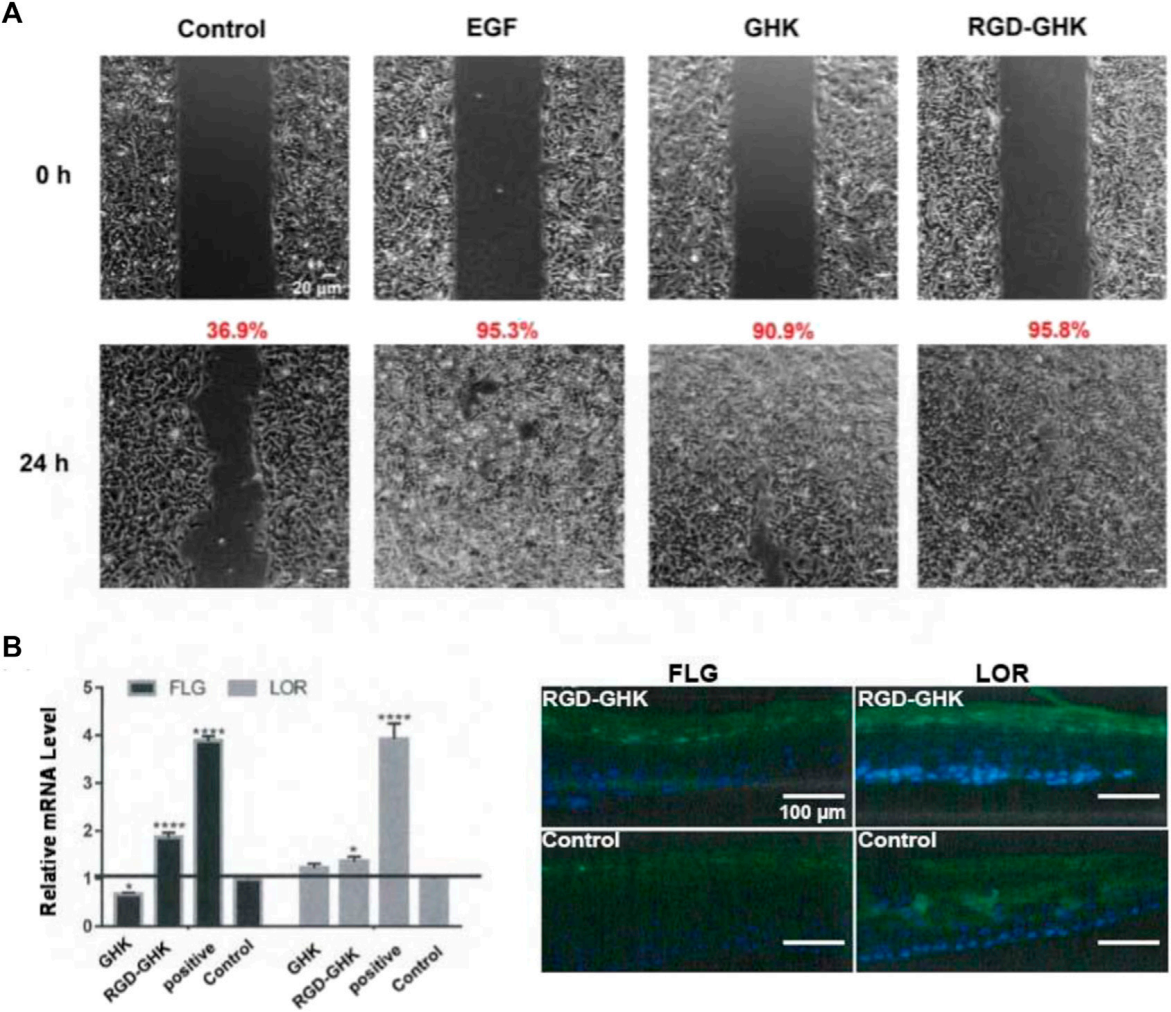
Effects of GHK and RGD-GHK on the closure of a wound field produced in a monolayer of HaCaT cells **(A)**. Cells were seeded on the two sides of an Ibidi culture insert and grown to 80% confluence. Afterward, cells were treated with 200 μg/mL of peptides or 10,000 IU/mL EGF. The control represents cells not treated with the peptide, as indicated. Cells were photographed at the time of insert removal (0 h) and evaluated for cell migration at 24 h from peptide addition. The percentage of the cell-covered area was calculated. Skin barrier repair of peptides in 3D skin model **(B)**. The skin was transferred into a 12-well plate and treated either with or without 2 mg/mL peptide at 37°C and 5% CO_2_ for 24 h. EpiSkin (human keratinocytes) was analyzed using qRT-PCR and immunofluorescence staining.

Data from our laboratory and others demonstrated that GHK has many biological effects on wound healing (Maquart et al., 1993), such as stimulation of collagen, glycosaminoglycan, and proteoglycan synthesis and acceleration of wound healing *in vivo*. Recently, Choi et al. (2012) showed a stem cell recovering effect of GHK in the skin, suggesting a modulating effect of this peptide on stem cell renewal. GHK and GHK-Cu peptides are widely used in cosmetic products for skin repair (Pickart and Margolina, 2018). In order to explore the skin repair effect of RGD-GHK, we first used an *in vitro* cell scratch assay to examine the healing effects of peptides and a positive control epidermal growth factor (EGF). Compared with background healing, treatment with GHK and RGD-GHK showed an obvious wound-healing rate of 90.9% and 95.8% at 24 h, respectively, while the control showed a rate of 36.9% at 24 h. RGD-GHK promoted the rate of wound repair to the same extent as EGF. GHK and GHK-derived targeting peptides facilitated scratch repair by accelerating collagen synthesis and glycosaminoglycan secretion (Figure 6A).

The aforementioned results suggested that the GHK-derived peptides exerted their anti-oxidative, anti-aging, and wound-healing activities in skin cells. We used a 3D skin model to further evaluate the repair activity of peptides. RGD-GHK significantly promoted the mRNA level of loricrin (LOR) and filaggrin (FLG), which were associated with a marked reduction in the damaged skin barrier. In Figure 6B, immunofluorescence staining of 3D skin showed that RGD-GHK induced 2-fold upregulation of FLG and LOR proteins compared to the control and GHK. Amphiphilic cell-penetrating peptide (CPP)-conjugated GHK was used as a positive control, which dramatically enhanced FLG and LOR expression.

### Proteolytic stability and hemolytic toxicity

**FIGURE 7:**
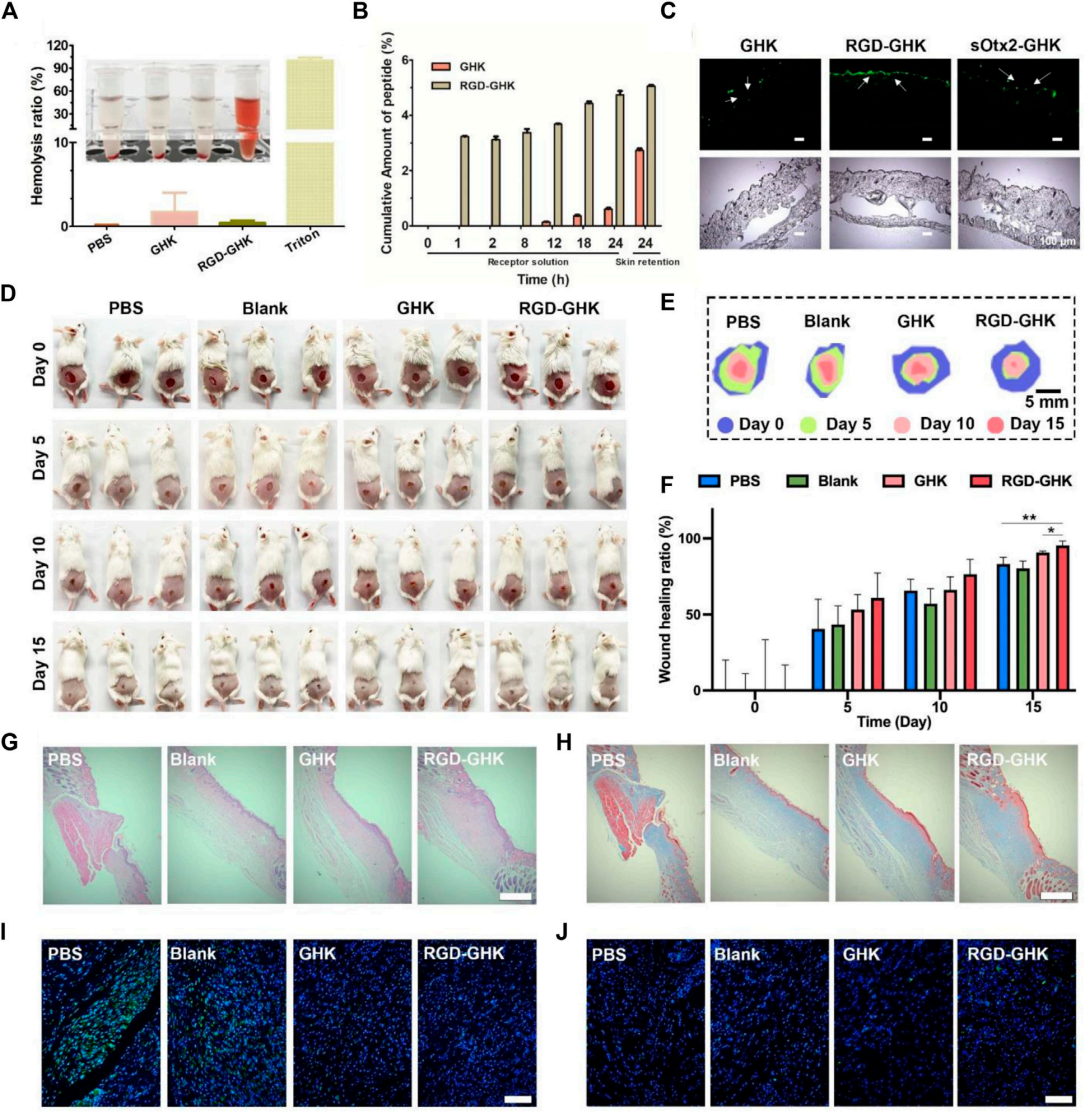
Wound healing effects *in vivo* of RGD-GHK. **(A)** Hemolytic activity of RGD-GHK. PBS and Triton X-100 (0.5%) were used as the negative and positive controls, respectively. **(B)** Permeation of GHK and RGD-GHK after 24 h *in vitro* skin permeation test with porcine skin. **(C)** Cell adsorption and penetration of peptide *in vivo*. **(D)** Pictures of the wound area on the 0th day, 5th day, 10th day, and 15th day for PBS, blank (cream), GHK (cream), and RGD-GHK (cream), and **(E)** traces of wound-bed closure during 15 days for each treatment. **(F)** Wound-healing ratio of each group during the treatment. H&E **(G)** and Masson’s trichrome **(H)** staining of the wound tissues. Scale bar: 250 μm. Representative photographs of skin wound tissues on the 15th day after immunofluorescence labeling with **(I)** IL-6 (green) and **(J)** CD31 (green). Scale bar: 100 μm.

Based on the aforementioned positive results, *in vitro* animal experiment was conducted to explore the potential of RGD-GHK in the practical cosmetics industry. First, we assessed the proteolytic stability of naked and modified GHK peptides. As depicted in Supplementary Figure S3, the conjugation of GHK with RGD made no significant difference in the proteolytic stability of the peptide. This indicates that the modification process does not compromise the stability of GHK. To evaluate the potential hemolytic toxicity of the peptide, we conducted hemolysis assays using red blood cells (RBCs). Figure 7A demonstrates that when incubated with the RGD-GHK peptide, less than 1% of the RBCs were lysed. The hemolytic activity of RGD-GHK peptide was comparable to that of the PBS buffer, suggesting minimal hemolytic toxicity of the modified peptide. This finding further supports the safety and biocompatibility of RGD-GHK in potential applications.

### Skin permeation *in vitro* and *in vivo*

The transdermal delivery of GHK and RGD-GHK was measured through the porcine skin. Permeation experiment was carried out for 24 h at 37 °C with Franz-type diffusion cells. Peptides in collected receptor solutions and skin retention were analyzed by HPLC. The results (Figure 7B) showed that approximately 5% of RGD-GHK and 0.5% of GHK were detected in the receptor cell, and 5% of RGD-GHK and 2.7% of GHK were detected in the skin. The results implied that the modification of RGD contributed to the skin adsorption and permeation of GHK. To further test whether the incorporation of a targeting motif could enhance the binding of chimeric peptides *in vivo*, we used smearing FITC-peptides at the mouse back for 24 h and tracked the peptide location of the skin slices by fluorescent microscopy. Figure 7C shows that most of RGD-GHK was concentrated on the epidermal layer, while only a few GHK and sOtx2-GHK were observed on the skin layer. The results illustrated that the RGD motif enhanced GHK adsorption and accumulation in the skin, which contributed to the local peptide density and the bioactivities of GHK. Here, although sOtx2-GHK exhibited better cell adhesion, its skin adsorption is weaker than RGD-GHK. One reason for the contradictory results of RGD-GHK binding *in vitro* and *in vivo* could be that high-molecular-weight peptide hindered the penetration and retention of sOtx2-GHK in the skin.

### Skin wound healing *in vivo*

The aforementioned results encouraged us to sequentially evaluate their application *in vivo*. As shown in Figure 7D, the wound area of all groups gradually decreased over a period of 15 days, and the RGD-GHK group exhibited the best wound-healing performance. The stacked placement results in Figure 7E further confirmed the superior capacity of RGD-GHK in promoting wound healing. Specifically, on day 5, the RGD-GHK group reduced the wound area by approximately 60.9% compared to day 0 (Figure 7F). The wound-healing speed not only surpassed that of the PBS (40.6%) and blank (43.4%) groups but also showed superiority over the GHK (53.3%) group on day 5. After a 15-day treatment, the wounds in the RGD-GHK (95.4%) and GHK (90.8%) groups were nearly closed, while a few wounds remained in the PBS (83.1%) and blank (80.3%) groups. This indicates that the introduction of GHK promotes wound healing, and the addition of cell surface-reactive cell-targeting peptide (RGD) enhances the wound-healing process of GHK.

Hematoxylin and eosin (H&E) staining was performed to confirm tissue regeneration in the wound area. Figure 7G shows that the wounds treated with RGD-GHK closed much faster compared to other groups. Additionally, Masson’s trichrome staining was used to assess the total collagen level in the wound site, which serves as a main marker of the wound-healing process. On the 15th day, the tissue in the wound area of the GHK and RGD-GHK groups exhibited a deeper blue color compared to other two groups, indicating higher collagen levels (Figure 7H). In summary, the H&E and Masson’s trichrome staining results support the conclusion that GHK possesses a good wound-healing promotion capacity. Furthermore, this ability is further enhanced by the introduction of a cell surface-reactive cell-targeting peptide in the peptide chain (RGD-GHK).

Interleukin 6 (IL-6) is a cytokine that plays a key role in regulating inflammation, immune response, acute phase response, and hematopoiesis, and IL-6 content is closely related to the degree of inflammatory response. To evaluate the total IL-6 level in the wound site, immunofluorescence labeling was performed. As shown in Figure 7F, the control group exhibited higher expression of IL-6 on the 15th day, indicating more severe inflammatory responses compared to other three groups. This can be attributed to the efficient inherent anti-oxidative ability of GHK, which helps reduce inflammatory response. In particular, the RGD-GHK group showed the lowest level of IL-6, suggesting that the introduction of RGD further enhances the anti-inflammatory properties of GHK. Platelet endothelial cell adhesion molecule-1 (CD31) is involved in angiogenesis and promotes wound healing. The levels of CD31 in the wound tissue increased gradually from the PBS, blank, and GHK groups to RGD-GHK groups on the 15th day. This indicates that GHK promotes wound healing by facilitating angiogenesis, and the introduction of RGD strengthens this capability. Overall, the inherent anti-oxidative ability of GHK promotes wound healing by reducing inflammatory response, and this ability is further enhanced by the introduction of cell surface-reactive cell-targeting peptide. The faster wound healing observed in the RGD-GHK group can also be attributed to faster angiogenesis.

## Conclusion

In summary, we successfully incorporated two cell-targeting motifs into tripeptide-1 (GHK), a well-known cosmetic peptide, resulting in the development of two chimeric peptides, namely, RGD-GHK and sOtx2-GHK. These cell-targeting motifs enhanced the interaction between GHK and oxidative skin cells, consequently improving the anti-oxidative, anti-apoptotic, and anti-aging properties of peptides. Additionally, the chimeric peptides demonstrated enhanced skin adsorption and penetration, excellent biocompatibility, and effectively facilitated *in vivo* wound healing. Overall, the optimized tripeptide-1 holds great potential as a novel and efficacious ingredient in cosmetics, warranting further exploration in cosmetic formulation and clinical trials.

## Data Availability

The original contributions presented in the study are included in the article/Supplementary Material; further inquiries can be directed to the corresponding author.

## Abbreviations

CS, chondroitin sulfate; caspases-3, cysteinyl aspartate-specific proteinase-3; ECM, extracellular matrix; FLG, filaggrin; GAGs, glycosaminoglycans; HaCaT, human keratinocyte; HFF-1, human fibroblast; Hyp, hydroxyproline; LDH, lactate dehydrogenase; LOR, loricrin; MMP-1, matrix metalloproteinase-1; RGD, Arg–Gly–Asp; ROS, reactive oxygen species.
