# Supplementary material for: Role of peptide–cell surface interactions in cosmetic peptide application
Source: Front Pharmacol. 2023 Nov 13;14:1267765. doi: 10.3389/fphar.2023.1267765 (PMC10679740; doi:10.3389/fphar.2023.1267765)

**Supporting Information**


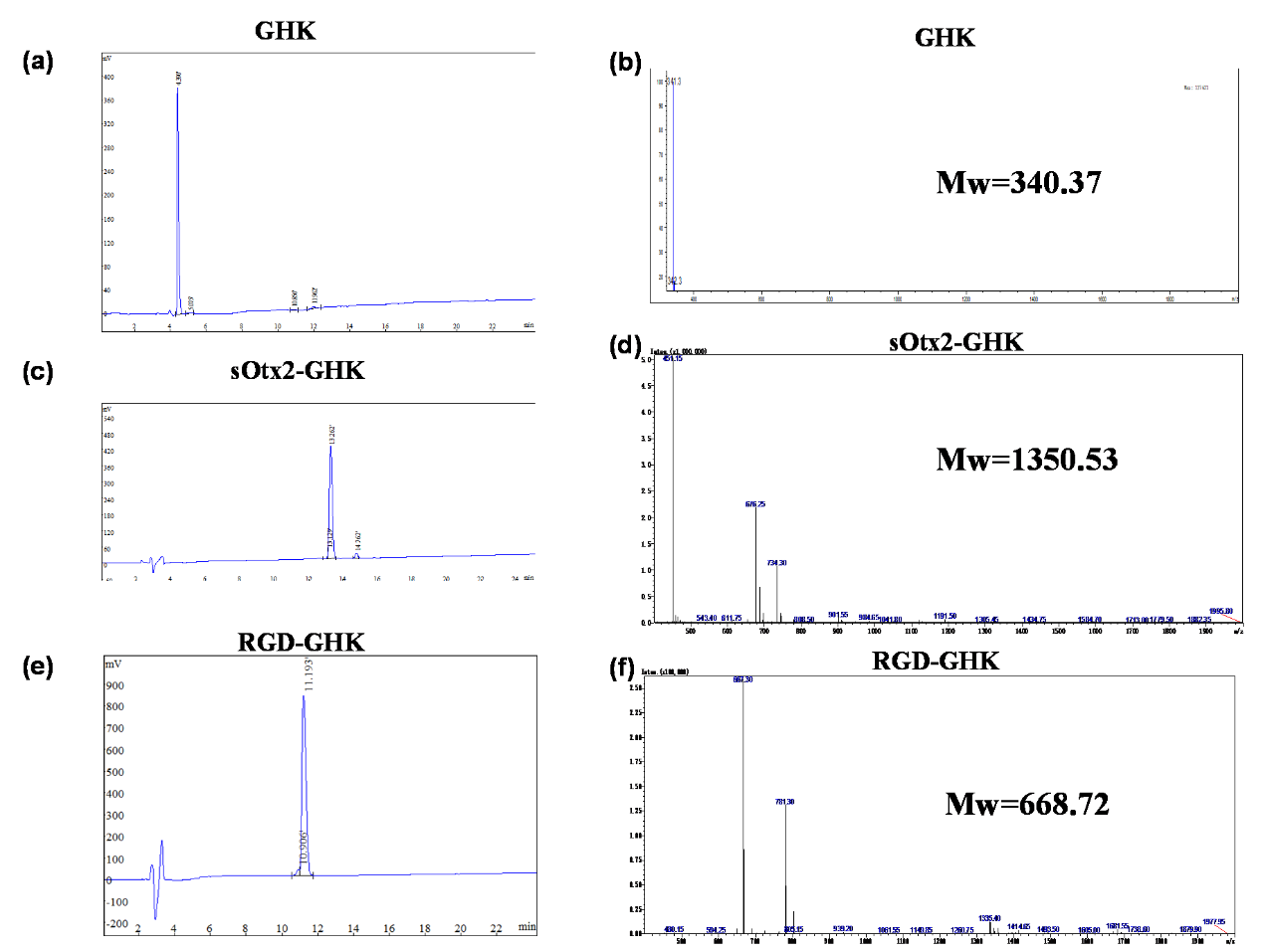


Figure S1. Quality control of GHK (a, b), sOtx2-GHK (c, d) and RGD-GHK (e, f) by mass spectrometry and HPLC.


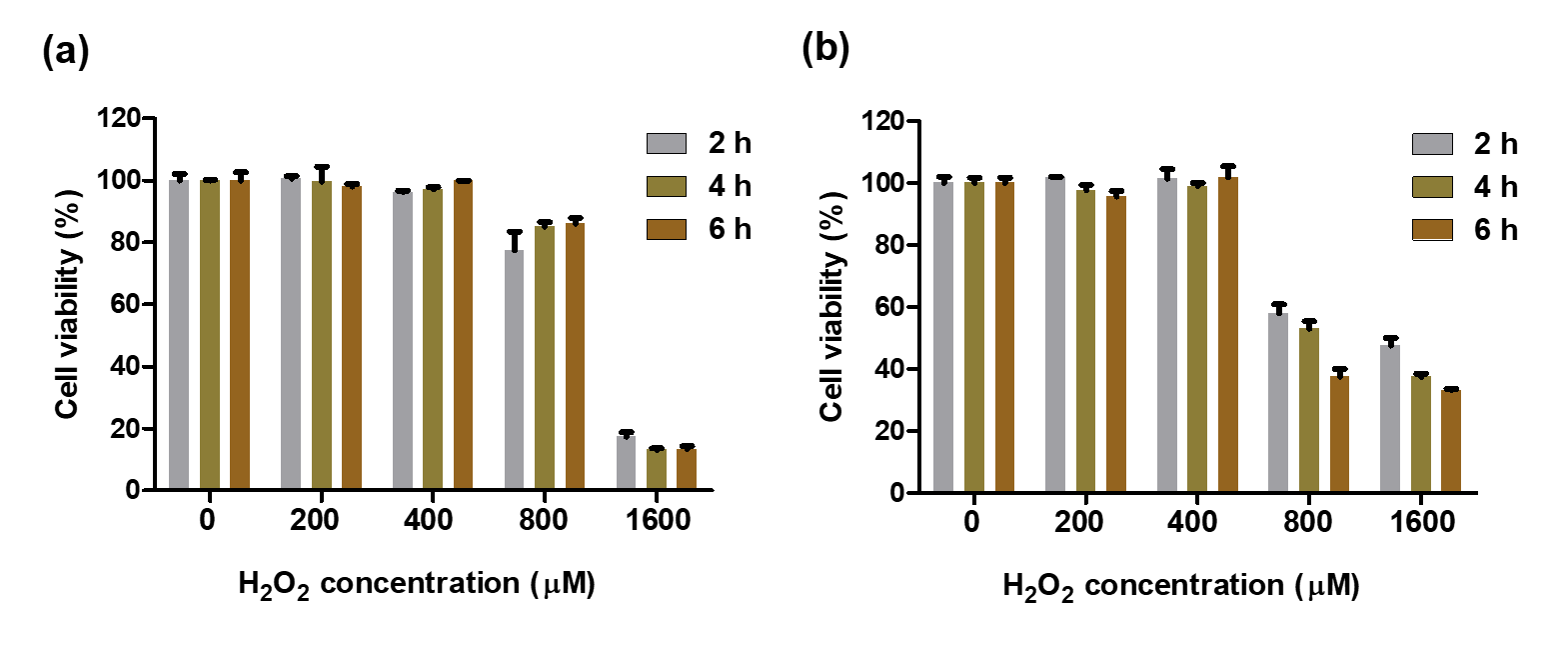


Figure S2. Cell viability of HaCaT (a) and HFF-1 (b) incubated with a range of concentrations of H_2_O_2_ for various incubation times.


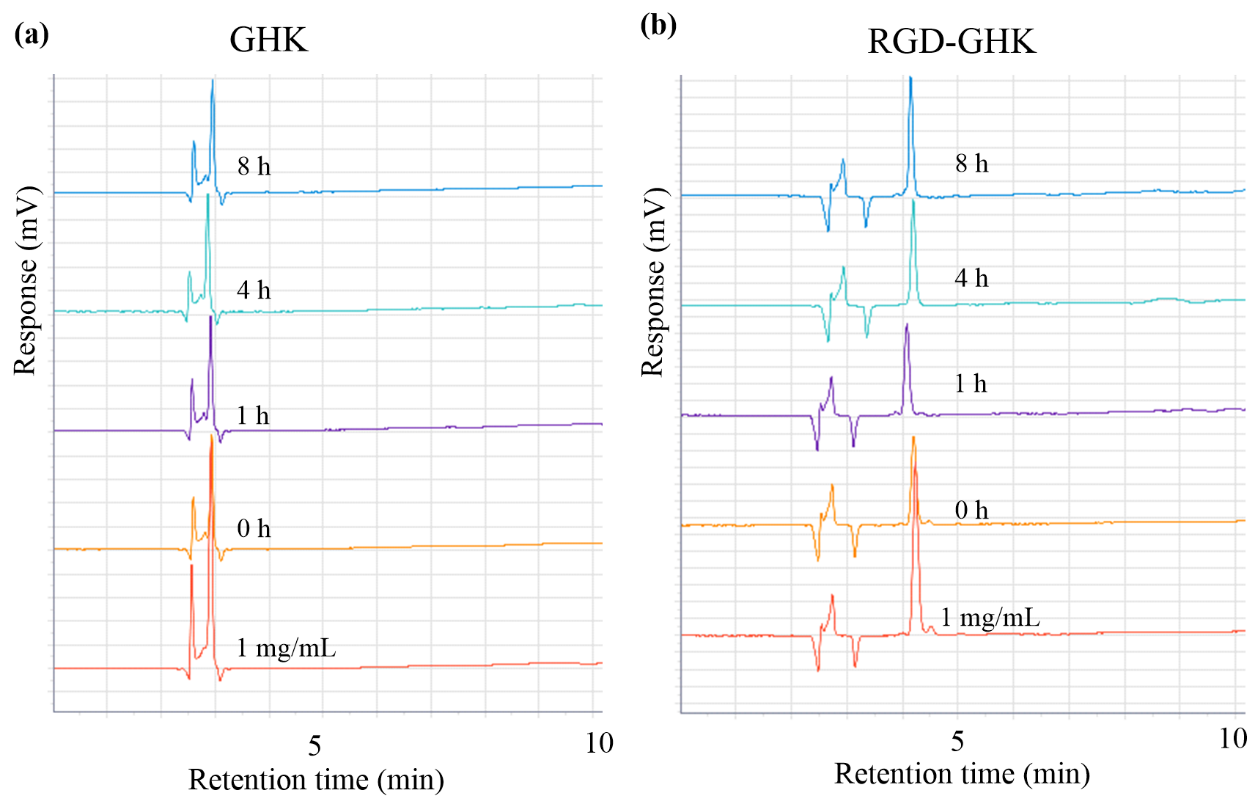


Figure S3. Proteolytic stability of GHK and RGD-GHK. HPLC spectra of GHK (a) and RGD-GHK (b) after incubation with chymotrypsin for 0-8 h.


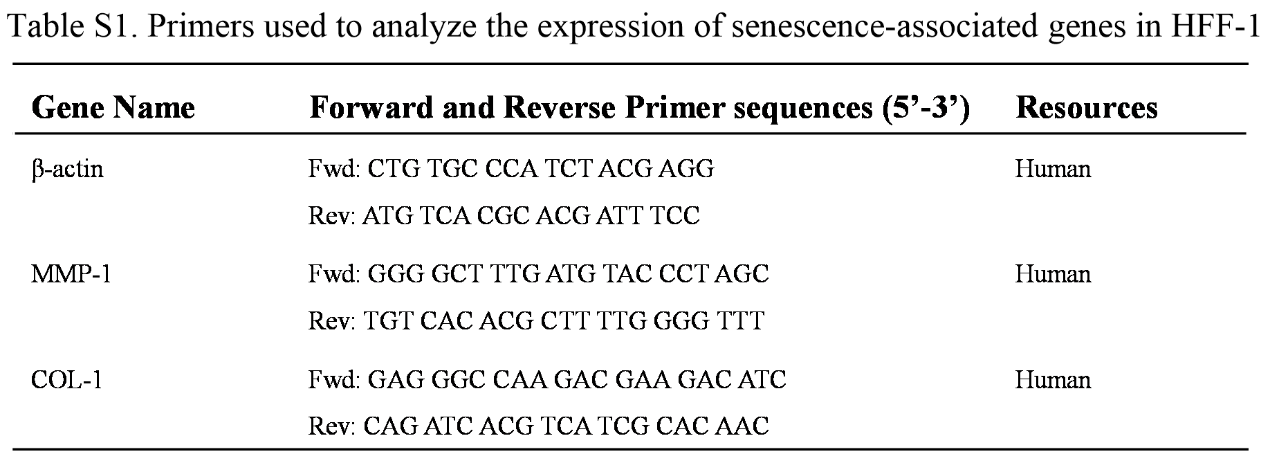

Supplement: Supplementary file 1 [file Table1.DOCX]
